# CD36 neutralisation blunts TLR2-IRF7 but not IRF3 pathway in neonatal mouse brain and immature human microglia following innate immune challenge

**DOI:** 10.1038/s41598-023-29423-0

**Published:** 2023-02-09

**Authors:** Shruti Gururaj Gadagkar, Melanie Lalancette-Hébert, Sai Sampath Thammisetty, Zinaida S. Vexler, Jasna Kriz

**Affiliations:** 1 CERVO Brain Research Centre, Université Laval, 2601 Chemin de la Canardière, Quebec, QC G1J2G3 Canada; 2 Department of Psychiatry and Neuroscience, Faculty of Medicine, Université Laval, 2601 Chemin de la Canardière, Quebec, QC G1J 2G3 Canada; 3 Department of Neurology, University California San Francisco, San Francisco, CA 94158 USA

**Keywords:** Neuroscience, Neuroimmunology

## Abstract

Innate immune response in neonatal brain is associated with a robust microglial activation and induction of Toll-like Receptors (TLRs). To date, the role of the scavenger receptor CD36 in TLRs modulation, particularly TLR2 signaling, has been well established in adult brain. However, the crosstalk between TLR4, TLR2 and CD36 and its immunogenic influence in the neonatal brain remains unclear. In this study, using a CD36 blocking antibody (anti-CD36) at post-natal day 8, we evaluated the response of neonates to systemic endotoxin (lipopolysaccharide; LPS) challenge. We visualized the TLR2 response by bioluminescence imaging using the transgenic mouse model bearing the dual reporter system luciferase/green fluorescent protein under transcriptional control of a murine TLR2 promoter. The anti-CD36 treatment modified the LPS induced inflammatory profile in neonatal brains, causing a significant decrease in inflammatory cytokine levels and the TLR2 and TLR3 mediated signalling.The interferon regulatory factor 3 (IRF3) pathway remained unaffected. Treatment of the LPS-challenged human immature microglia with anti-CD36 induced a marked decrease in TLR2/TLR3 expression levels while TLR4 and IRF3 expression was not affected, suggesting the shared CD36 regulatory mechanisms in human and mouse microglia. Collectively, our results indicate that blocking CD36 alters LPS-induced inflammatory profile of mouse and human microglia, suggesting its role in fine-tuning of neuroinflammation.

## Introduction

Inflammation is a key component of the innate immune response. Primarily designed to remove noxious agents and limit their detrimental effects, the prolonged and/or inappropriately scaled innate immune response may be detrimental to the host and lead to a disease^1,2^. Microglia are the principal immune cells of the brain. Once activated, they robustly express and/or induce pattern recognition receptors (PRRs) such as TLRs. PPRs sense pathogen-associated molecular patterns as well as endogenous danger-associated molecular pattern ligands associated with sterile inflammation observed in brain injuries and stroke^3,4^. At present, the role of different TLRs in the context of brain injury and/or infection has been well established in the adult brain^5^. While TLR4 is mostly injurious in both experimental and human stroke, owing to its versatile heterodimerization, the role of TLR2 is more complex and it can elicit both injurious and beneficial context- and tissue-dependent effects^6–11^. To date, several research groups, including ours, have described the immune cascades following the activation of microglial TLR2 in neonates^4,12–16^. By using TLR2 reporter mouse model and in vivo bioluminescence imaging approach, we recently showed that under physiological conditions, TLR2-luc is highly induced in the early postnatal brain and undergoes a marked ∼30-fold decline during the second and third postnatal weeks, suggesting distinct dynamics mechanisms for innate immune signalling and homeostasis in the immature brain^4,16^.

TLRs are known to interact with different co-receptors. Cluster of differentiation 36 (CD36) is one of the well described TLRs interacting partners, with preference to create heterodimers with TLR2. CD36 is a surface glycoprotein present in a variety of cells, including microglia, macrophages, endothelial cells, cardiac and skeletal muscle, adipocytes, and platelets^9^. Although CD36- affected pathways are well characterized in adults, its role in regulating immunity during the neonatal period remains elusive. For example, genetic deletion of CD36 offers protection against acute injury after middle cerebral artery occlusion (MCAO) in adults, whereas it leads to more severe ischemic injury in neonates^17^. Evidence suggests that the effects of genetic deletion of CD36 in acute neonatal stroke are, in part, due to the diminished phagocytosis of apoptotic neurons in injured neonatal brain and the consequent accumulation of the caspase-3 positive cells^18^. Furthermore, CD36 toxicity may occur due to a marked increase in superoxide production caused by NADPH oxidase (Nox) activation. However, the contribution of individual Nox isoforms and consequent superoxide utilization in injured brain differs in neonatal stroke^19^. Indeed, superoxide accumulation is observed in microglia/macrophages in the acutely injured adult but not in neonatal brain^17,18,20^.

Given the distinct role of CD36 and innate immune signalling in neonatal brain, in the current study, we further explored the role of scavenger receptor CD36 in regulation of immune profiles in neonatal brain microglia/macrophages as well as in human embryonic microglia. By using a specific CD36 receptor blocking antibody in conjunction with biophotonic/bioluminescence imaging in the live TLR2-luc GFP mice, here we show that systemic delivery of an anti-CD36 antibody completely blunts TLR2 induction following LPS-mediated innate immune challenge in P9 brain. Furthermore, we show that the acute inhibition of CD36 receptor before systemic LPS administration significantly affects TLR2 and TLR3 downstream signalling while TLR4-IRF3 pathway remains relatively spared in the neonatal mouse brain. Importantly, the treatment of embryonic human microglial cell line HMC3 with anti-CD36 blocking antibody exerts similar regulatory effects on TLR2 and TLR3 expression patterns, while leaving TLR4-IRF3 expression/signalling unaffected. Together, our results suggest a role for CD36 in the fine-tuning of neuroinflammation in immature mouse brain and in immature human microglia.

## Results

### Pre-treatment with CD36 blocking antibody blocks in vivo induction of the TLR2 bioluminescence signal

**Figure 1:**
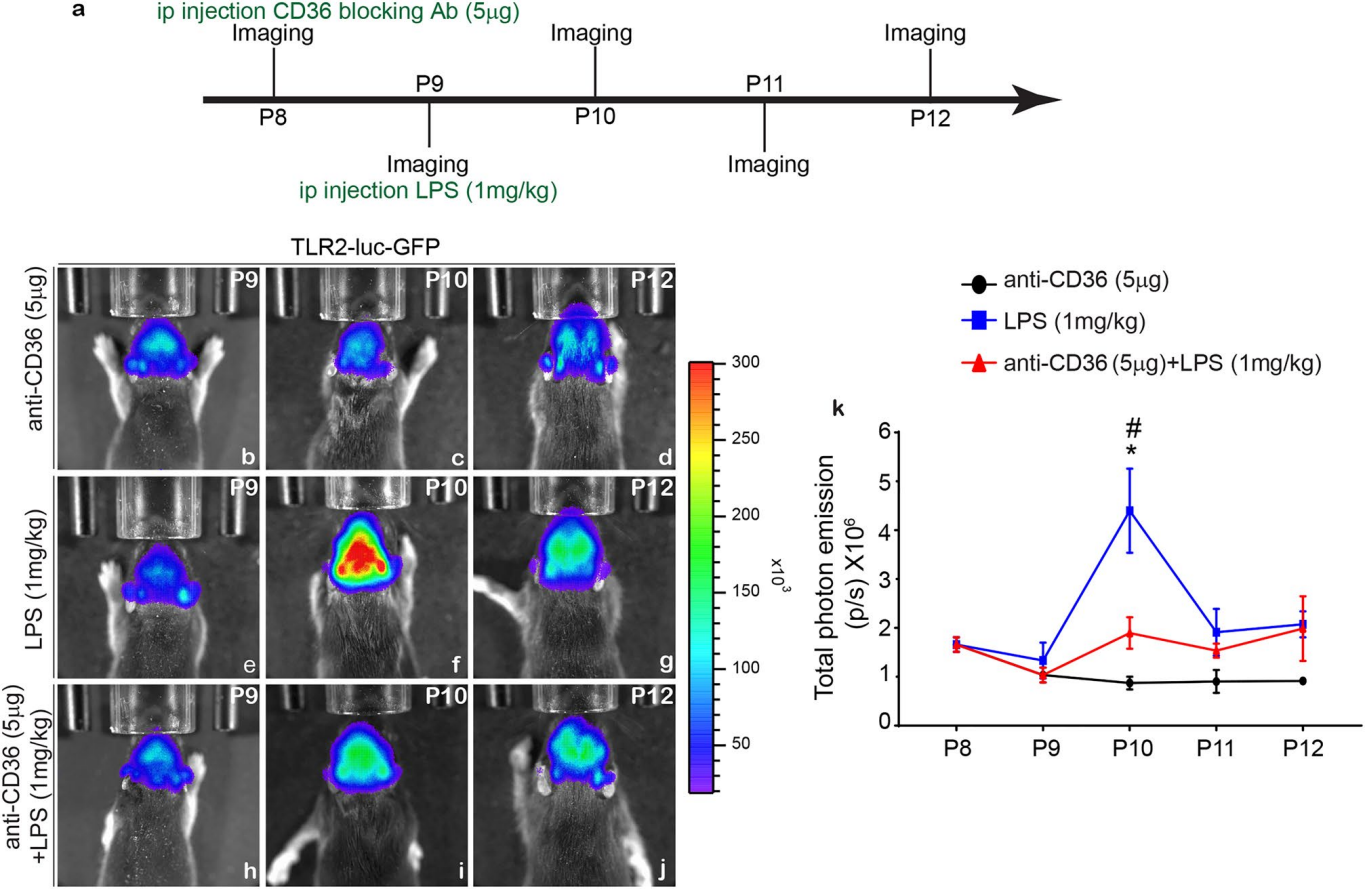
Real-time bioluminescence imaging of theTLR2 signals after LPS challenge. (**a**) Schematic presentation of experimental protocol used for the treatment with anti-CD36 antibodies in P8-P9 mice. (**b**–**j**) Representative images of the bioluminescent signals recorded from the brains of living P10–P12 TLR2-luc-GFP mice treated with anti-CD36 antibody (**b**–**d**), LPS (**e**–**g**) or pre-treated with anti-CD36 antibody followed by systemic LPS injection (**h**–**j**). Note that a robust TLR2 induction 24 h post-LPS treatment is significantly reduced when the pups are pre-treated with anti-CD36. The scales on the right are the colour maps for source intensity. The scale ranges differ at individual ages. (**k**) Quantification of the in vivo bioluminescence data from P8–P12 (in photons per second, p/s). One way ANOVA (anti-CD36 vs LPS *p ≤ 0.05, LPS vs anti-CD36 + LPS *p ≤ 0.05).

We previously reported that genetic deletion of CD36 dysregulates TLR2 expression following tMCAO in neonatal mice^21^. Here we investigated the contribution of co-receptors CD36, TLR2, to the overall inflammatory response and innate immune signalling in microglia/macrophages in living neonatal brain. The innate immune challenge was induced by systemic LPS administration. To assess the effects of CD36, we used an anti-CD36 blocking antibody delivered i.p. to P8 mice, 24 h prior to systemic LPS challenge. Next, we took advantage of the TLR2-luc-GFP mouse line previously generated and validated in our laboratory^4,16^. In this mouse model, co-expression of the reporter genes luciferase (and GFP) under the transcriptional control of the murine TLR2 gene promoter allows a real time visualization of the luciferase signals from the brains of living mice, through biophotonic/bioluminescence imaging^4,16^. Our previous analyses revealed that the systemic LPS injection stimulates TLR2 induction/signalling and induces synthesis and/or secretion of inflammatory mediators in the brains of P6–P9 mice^4^. As schematically presented in Fig. 1a, the TLR2-luc-GFP pups were first treated with CD36 neutralizing antibody (i.p.5 μg) at P8 followed by LPS at P9 using dosing established in our preliminary experiments and previously published studies^4,16^. As expected, we observed a significant increase in the TLR2 signal 24 h post LPS treatment when compared to controls (Fig. 1b–k). In contrast, TLR2 signal induction was blunted in the brain of mice pre-treated with the CD36 neutralizing antibodies (Fig. 1h–j). Quantitative analysis of total photon emission counts revealed a twofold decrease in the TLR2 signal intensities in the LPS challenged mice pre-treated with CD36 neutralizing antibody (Fig. 1k), thus suggesting that blocking scavenger receptor CD36 antagonizes LPS-induced TLR2 induction in the neonatal brain (Supplementary Information).

### CD36 neutralizing antibody arrests TLR2 activation and silences microglia

**Figure 2:**
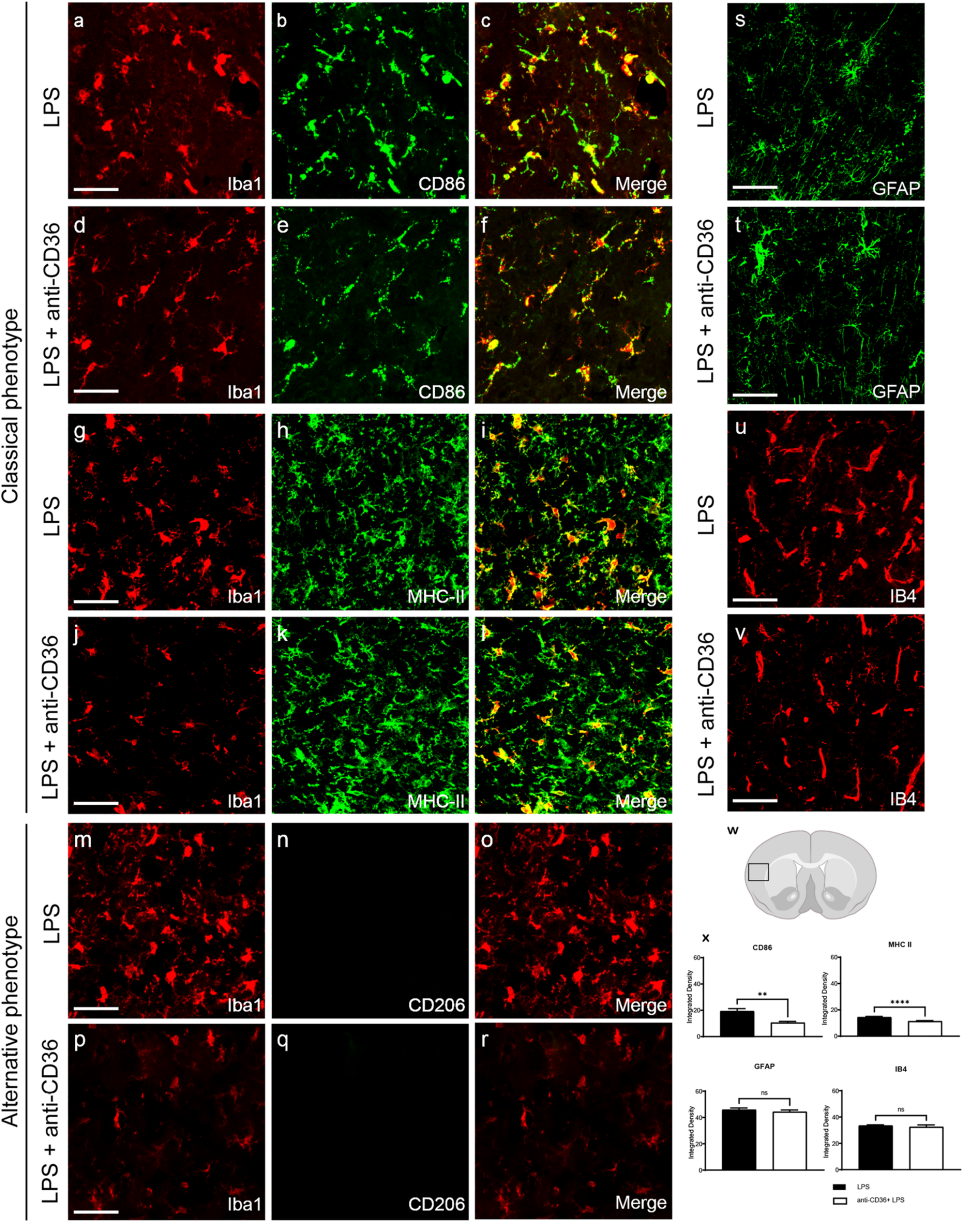
Neonatal microglia demonstrate a reduced classical phenotype when treated with anti-CD36 antibody. (**a**–**v**) Immunostaining of neonatal brain sections with classical and alternative microglia phenotypic markers (**a**–**r**), with astrocyte marker GFAP (**s**,**t**) and IB4 (**u**,**v**). (**w**) Area of the cortical section analysed and quantification of CD68, MHC II, GFAP and IB4 expression (**x**). Microglial cells (Iba1 +) show increased expression of the classical activation markers CD86 (**a**–**f**) and MHCII (**g**–**l**) after systemic LPS injection (x). Their expression is significantly diminished when the pups are pre-treated with anti-CD36 antibody (**d**–**f** and **j**–**l**). Quantification of the signals in arbitrary units shows an overall decrease in expression of the classical markers when the pups are injected with anti-CD36 before the LPS treatment (**x**). Microglia in both the groups did not express the alternative microglial activation marker CD206 (**m**–**r**). No significant decrease was observed with the astrocytic marker GFAP in LPS and anti-CD36 + LPS treated pups (**s**,**t**,**x**). A non-significant decrease in the expression of IB4 is observed in the anti-CD36 + LPS pups when compared to the LPS treated pups (**u**,**v**,**x**). Scale bar is 100 µm. Unpaired t test with Welch's correction ***p ≤ 0.001; **p ≤ 0.01.

Although CD36 neutralizing antibody was delivered systemically, the above-described in vivo imaging results suggest an immune modifying effect on the brain microglia. Indeed, our previous work revealed that in TLR2-luc-GFP mice a standard systemic LPS challenge is associated with a marked induction of the TLR2 signal in microglial cells in adult as well as neonate mice^4,16^. To assess the effects of CD36 neutralizing antibody on microglial phenotypes, we examined expression of standard markers used for functional differentiation of classical inflammatory vs alternative microglia, including CD83, MHCII, Iba1 and CD206. As shown in Fig. 2, double immunofluorescence analyses revealed a marked increase in immunostaining for markers CD86 (Fig. 2a–c) and MHCII (Fig. 2g–i) in Iba1 + positive cells in LPS treated mice. Treatment with CD36 neutralizing antibody reduced LPS-mediated increase of CD86 (Fig. 2d–f) (p < 0.0001) and MHCII (p = 0.0012) (Fig. 2j–l) immunoreactivity in Iba1 + cells. On the other hand, we did not observe changes in expression patterns of the alternative microglial marker CD206 (Fig. 2m–r), as the neonatal Iba + cells were devoid of any CD206 immunoreactivity in the tested experimental conditions. Further analyses revealed no significant change in expression of IB4 in the CD36 + LPS group when compared to LPS alone (Fig. 2u,v,x). Of note, we did not observe differences in expression patterns of the astrocytic marker GFAP in LPS and CD36 + LPS treated group (Fig. 2s,t,x), suggesting that following acute systemic LPS challenge, CD36-mediated effects preferentially target microglia. Overall, the classical activation of microglia following LPS was attenuated by pre-administration of the CD36 neutralizing antibody.

### Blocking TLR2 signalling by anti-CD36 antibody reduces expression of pro-inflammatory cytokines in neonatal mouse brain

**Figure 3:**
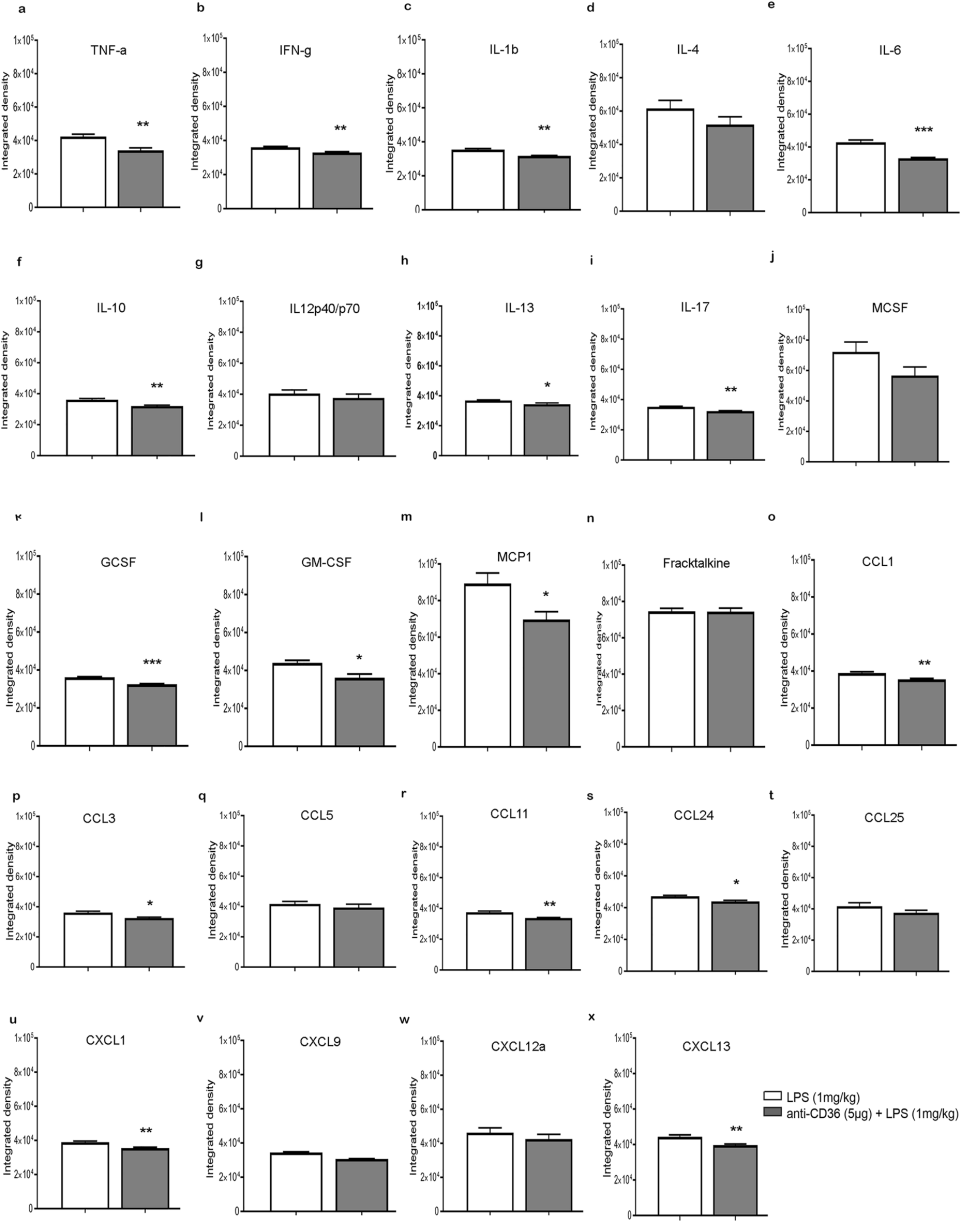
Treatment with anti-CD36 antibody reduces expression of inflammatory mediators after LPS challenge. (**a**–**x**) Protein expression of 24 inflammatory cytokines reveals a significant reduction in cytokines (**a**) TNF-a, (**b**) IFN-g, (**c**) IL-1b, (**e**) IL-6, (**f**) IL-10, (**h**) IL-13, (**i**) IL-17, along with (**k**) GCSF, (**l**) GM-CSF, (**m**) MCP-1 and chemokines (**p**) CCL3, (**r**) CCL11 (**s**) CCL24, (**u**) CXCL1, (**v**) CXCL9 and (**x**) CXCL13 when the pups are treated with anti-CD36 before LPS. Unpaired t-test ***p ≤ 0.001; **p ≤ 0.01 and *p ≤ 0.05.

Next, we analyzed the effects of the CD36 neutralizing antibody on the expression patterns of different immune mediators in the neonatal mouse brain. Previously, we reported that following systemic LPS treatment in neonatal mice, there is a significant increase in expression of pro-inflammatory cytokines, along with modest increase in the levels of several anti-inflammatory and growth factors^16^. In the current study,, we measured the protein expressions of 22 different immune mediators including, cytokines, chemokines and different colony stimulating factors 24 h after LPS challenge at P9, with and without anti-CD36 antibody pre-treatment. As shown in Fig. 3 the normalised densitometry measurements revealed a significant decrease in the levels of pro-inflammatory cytokines, including TNF-a (p = 0.0071), IL-1b (p = 0.0014), IFNg (p = 0.007), IL-6 (p = 0.0006), IL-17 (p = 0.0013). We also noted a decrease in the levels of anti-inflammatory cytokines such as IL 10 (p = 0.007) (Fig. 3f) and IL-13 (Fig. 3h) (p = 0.0293). In addition, expression levels of the colony growth factors G-CSF (Fig. 3k) (p < 0.0001), GM-CSF (Fig. 3l) (p = 0.0153), as well as different chemokines including MCP-1 (Fig. 3m) (p = 0.0333), CCL3 (Fig. 3p) (p = 0.0161), CCL11 (Fig. 3r) (p = 0.0037), CCL24 (Fig. 3s) (p = 0.0104), CXCL1 (Fig. 3u) (p = 0.0047), CXCL9 (Fig. 3v) (p = 0.0002) and CXCL13 (Fig. 3x) (p = 0.0075), were also significantly reduced. Hence, our results suggest that blocking CD36 hampers the release of both pro-and anti-inflammatory cytokines as well as chemokines and growth colony factors, suggesting an overall suppression of immune signalling in immature brain.

### CD36 neutralisation blunts TLR2-IRF7 but not IRF3 pathway in neonatal mouse brain following systemic LPS administration

**Figure 4:**
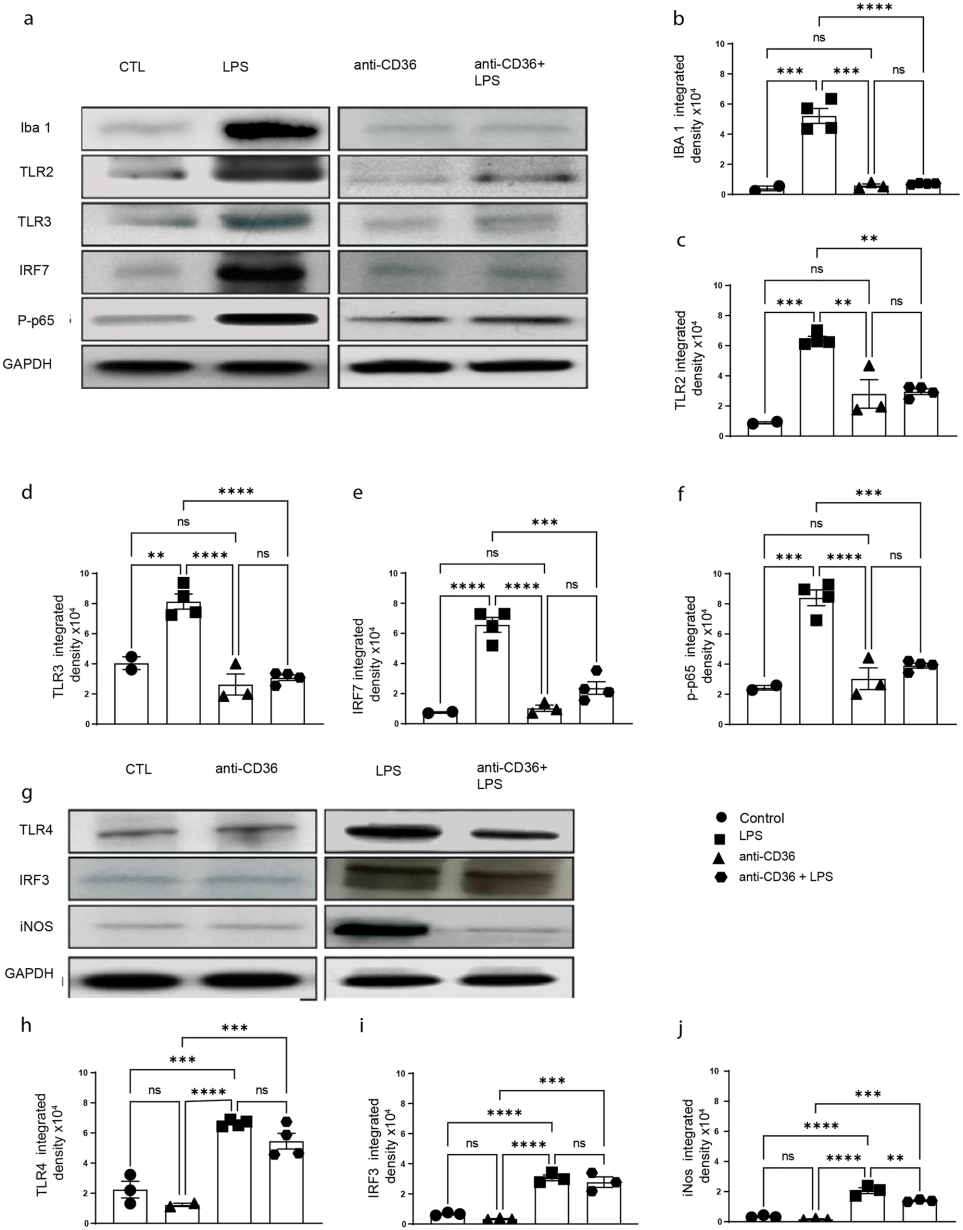
Blocking CD36 significantly alters TLR-2 and TLR-3 signalling. (**a**) Western blot of whole brain protein lysates from the control (saline), anti-CD36, LPS and anti-CD36 + LPS injected pups. (**b**) Iba1, (**c**) TLR2, (**d**) TLR3, (**e**) IRF7, (**f**) phosrpho-P65 expression levels were quantified in different conditions. GAPDH was used as loading control A significant increase in the levels of all the tested proteins is observed after LPS injection. Treating the pups with anti-CD36 before LPS injections considerably reduces their expression to the level as observed in the control pups. (**g**) Western blot of total protein lysates from the control, anti-CD36, LPS and anti-CD36 + LPS injected mice. (**h**) TLR4, (**i**) IRF3, (**j**) iNOS expression levels were quantified in above-described conditions. GAPDH is used as loading control. A significant increase in the levels of all the proteins tested is observed after LPS injection when compared to control and anti-CD36 treated mice. Of note, LPS-mediated increase in expression of TLR4/IRF3 is maintained in the anti-CD36 + LPS treated group. The entire data was presented as mean ± SEM and statistical significance between the groups was achieved using one-way ANOVA with Tukey’s multiple comparison test (ctl vs LPS, ctl vs anti-CD36, LPS vs anti-CD36 + LPS) and depicted as ****p ≤ 0.0001; ***p ≤ 0.001; **p ≤ 0.01 and *p ≤ 0.05.

To better understand the cellular and molecular mechanisms involved in the observed silencing of immune response, we next investigated the downstream pathways potentially affected by CD36 neutralization. It has been widely established that during activation of innate immune response CD36 may interact with various PPRs converging mainly on two major signalling pathways, NF-κB and IRF3^22^. LPS challenge is associated with robust induction of several signalling pathways downstream of TLR’s, including NF-κB, IRF3 and IRF7^16,23^. In order to investigate how anti-CD36 antibody mediated silencing of microglia affects innate immune signalling, we analysed expression patterns of different TLRs including TLR2, TLR3 and TLR4 (Fig. 4a,c,h) as well as their downstream signalling targets such as IRF3 and p-p65, iNOS and IRF7 (Fig. 4a,i,f,j,e). As expected, western blot analyses 24 h following LPS challenge revealed a significant increase in the expression levels of TLR2 and TLR3 (Fig. 4a–f), associated increase in expression of IRF7 and markers of activated form of NF-kB, p-p65. Expression levels of Iba1 were used as a general readout of microglia activation in the brain (p < 0.0001, F = 58.27). As further shown in Fig. 4, and in accordance with the results obtained in in vivo imaging experiments (see Fig. 1), treatment with CD36 neutralizing antibody completely blunted LPS-mediated increase in TLR2 (p = 0.0001, F = 23.85) and TLR3 (p < 0.0001, F = 32.11,) expression and associated increase in IRF7 (p < 0.0001, F = 41.85,), as well as p-p65 (p < 0.0001, F = 33.41) expression. Interestingly, western blot analyses revealed that treatment with an anti-CD36 neutralizing antibody exerted a modest effect on the expression of TLR4 (p < 0.0001, F = 33.55) and IRF3 (p < 0.0001, F = 48.56), suggesting that the TLR4-IRF3 pathway remains partially unaffected (Fig. 4g,h). This indicates that CD36 co-receptor plays an important role in TLR2 and TLR3-mediated inflammation without having a significant impact on TLR4-IRF3 pathway.

### Neutralizing CD36 affects TLR2 but not TLR4-IRF3 pathway in human immature microglia

**Figure 5:**
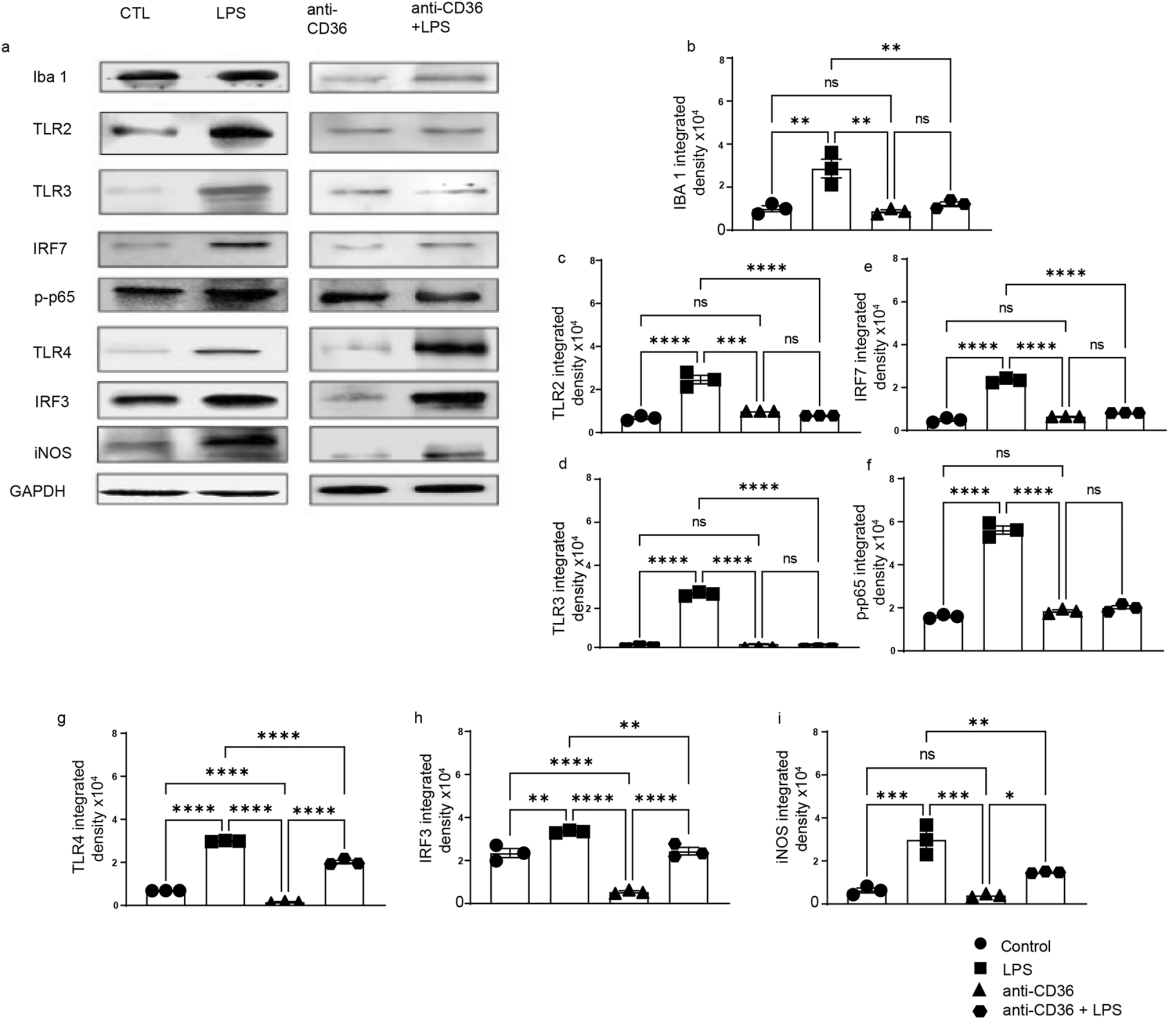
Blocking CD36 in human immature microglia affects the TLR3, TLR2-IRF7 but not TLR4/IRF-3 pathway. (**a**) Western blot of total protein lysates of HMC3 cells treated with anti-CD36 antibody, followed by LPS treatment after 24 h. Expression levels of (**b**) Iba-1, (**c**) TLR2, (**d**) TLR 3 along with (**e**) IRF 7, (**f**) p-P65, (**g**) TLR 4, (**h**) IRF-3, (**i**) iNOS were analysed and quantified. GAPDH was used as loading control. LPS treatment increases expression levels of all measured proteins while pre-treatment withanti-CD36 antibody prevented increase in their expression levels. Expressions of (**g**) TLR 4 and (**h**) IRF-3 (**i**) were not affected by anti-CD36 antibody pre-treatment.. Entire data in the figure was presented as mean ± SEM and statistical significance between the groups was achieved using one-way ANOVA with Tukey multiple comparison test and depicted as ****p ≤ 0.0001; ***p ≤ 0.001; **p ≤ 0.01 and *p ≤ 0.05.

**Figure 6:**
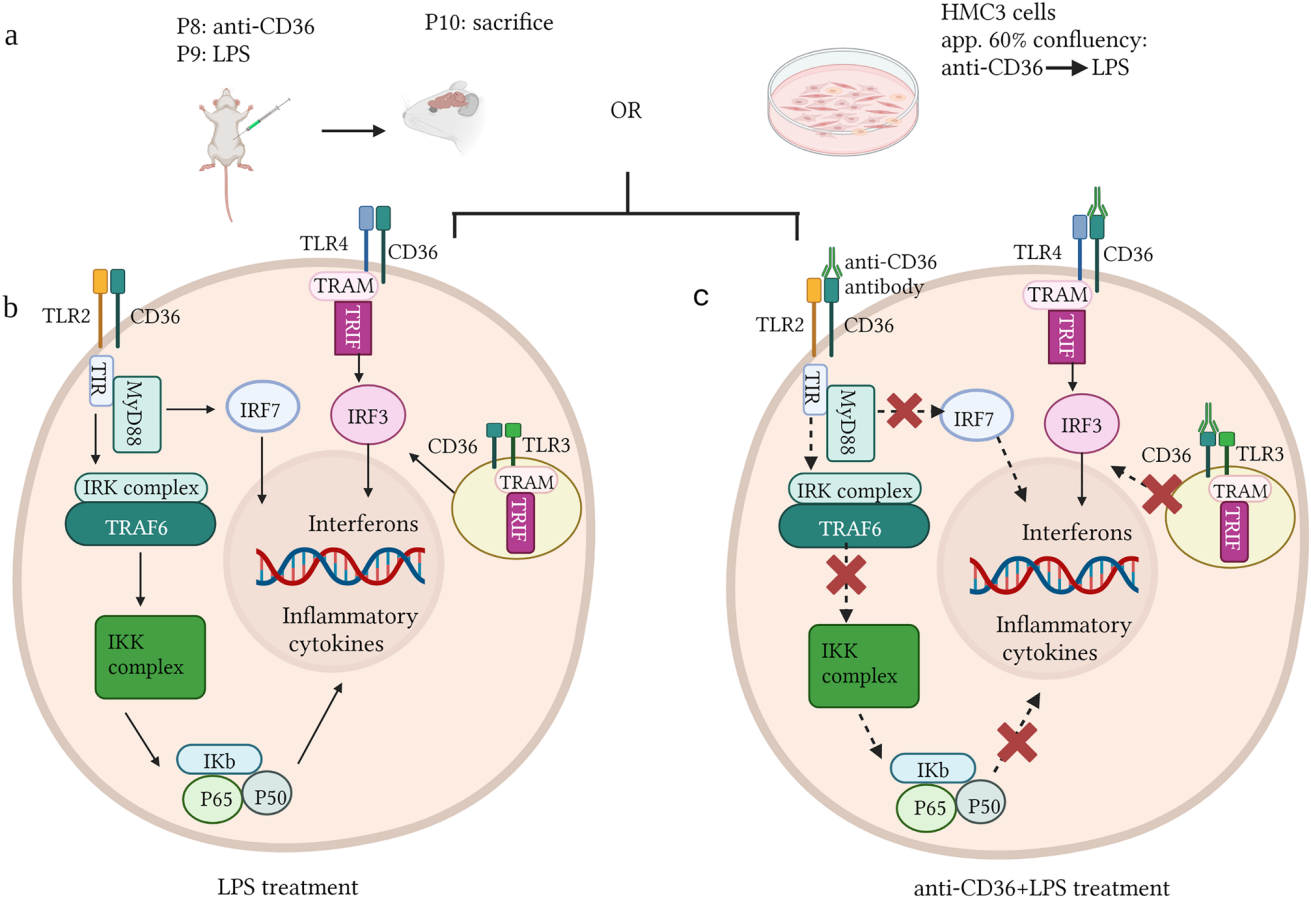
CD36 mediated-effects on TLR signalling pathways in neonatal brain and immature human microglia. A diagram showing the mechanism of the scavenger receptor CD36-TLR mediated signalling in neonatal mouse brain as well as human microglia. (**a**) schematic representation of the methodology used. Neonatal mice and HMC3 cells were treated with LPS or anti-CD36 + LPS. (**b**) Upon activation by various ligands, the TLR2-CD36 mediated MyD88 dependent pathway activates transcription of various inflammatory cytokines. Activated endosomal TLR3 activates IRF3, which promotes transcription of the genes related to type 1 Interferons. Interestingly, IRF3 is also activated by TLR4, through the TRAM-TRIFF complex mediated cascade. (**c**) Blocking CD36 with the antibody prior to the LPS stimulation affects the TLR2 and TLR3 mediated signalling while the TLR4-IRF3 pathway continues to contribute to inflammation in both neonatal mouse as well as in human microglial cells. The schematic was created with BioRender.com.

To validate translational potential of our findings, we used primary human microglia cells (HMC3 line) derived from the foetal brain and determined whether effects of the CD36 neutralizing antibody on TLR2 and TLR3 signalling can be reproduced in human immature microglia. Human microglia cells grown to ~ 60% confluency were either challenged by LPS or pre-treated with an anti-CD36 antibody followed by LPS challenge. As already described, the expression patterns of several PPRs and their respective downstream targets were analysed at protein level. Western blots analysis of human microglial cell lysates revealed that, as expected, LPS induces an over-expression of PPRs such as TLR2, TLR3 and TLR4, resulting in significant increase in the levels of p-p65, IRF7 and iNOS (Fig. 5a,c,d,f,i), as well an increase in expression levels of microglial marker Iba1(Fig. 5b). Pre-treatment with anti-CD36 neutralizing antibodies prevented LPS-mediated induction of TLR2 (p < 0.0001, F = 65.35), and TLR3 (p < 0.0001, F = 1811), resulting in reduced activation of transcription factors such as P-p65 (p < 0.0001, F = 276.8), IRF7 (p < 0.0001, F = 399.9) as well as iNOS (p < 0.0001, F = 31.67) (Fig. 5e,f,i). As further revealed in Fig. 5g,h, neutralizing CD36 antibody did not significantly affect expression levels of TLR4 (p < 0.0001, F = 949) and IRF3 (p < 0.0001, F = 69.19) suggesting that, as observed in the context of neonatal mouse model, the TLR4-IRF3 pathway remains unaffected after blocking CD36. Together, our results suggest a role of CD36 in regulation of innate immune response in mouse and human microglia. The proposed mechanism is presented in Fig. 6.

## Discussion

The innate immune response represents the first line of host defence against injury and/or infection in adult as well as in neonatal brain. Here, we provide an important in vivo and in vitro evidence for the role of CD36 in regulation of innate immune response after endotoxin (LPS) infection and TLRs signalling in neonatal brain as well as in immature human microglial cells. Selective inhibition of CD36 by using a neutralizing antibody prior to systemic innate immune challenge abolished acute LPS-mediated TLR2 induction observed in P10 mice and was accompanied by a generalized silencing of innate immune response as revealed by multiplex cytokine array analyses. Furthermore, disrupted CD36 signalling silenced TLR2 and TLR3 expression as well as the expression of their downstream targets such as IRF7. Although blocking CD36 neutralization reduced in part levels of TLR4, IRF3 expression remained unaffected. Importantly, we show here that CD36 carries the same regulatory role immune-modulatory potential in immature human microglia.

Microglia are the primary immune cells in the brain, continuously sensing novel stimuli, performing housekeeping and defence functions^24^. One of the hallmarks of microglial response to injury and/or pathogenic insult is a robust induction of several TLRs, including TLR2^25^. Scavenger receptors, such as CD36, aide in interactions between the TLRs and their ligands, thereby enabling further downstream signalling, which ultimately results in transcription of several pro-inflammatory cytokines and chemokines^26^. However, recent evidence suggests that the role of CD36-mediated immune signalling may differ in adult vs immature brain, as, contrary to the response to ischemic injury in adult brain, CD36 deficiency was associated with higher incidence of larger ischemic lesion in P9 pups during acute injury phase^18,20,21^. While in adult stroke, CD36-dependent NF-κB activation mediates injury, the role of NF-κB pathway in neonatal brain is more complex^17^. Early and transient pharmacological disruption of the IKK-NF-κB complex exerted certain level of protection, i.e., attenuated microglial activation and cytokine production after hypoxia–ischemia in neonates, whereas sustained inhibition of this pathway for 24 h was shown to aggravate injury^27,28^.

In the current study, we were able to further explore the role of CD36 -TLR2 interaction in the context of innate immune response in immature brain. By using real-time in vivo bioluminescence imaging in the TLR2-luc-GFP model, we found that blocking CD36 by using neutralizing antibodies significantly abolished the TLR2 signal induction following the LPS challenge, in the brains of living P9–10 mice. We also observed a marked downregulation of TLR2 and TLR3 expression, which consequently lead to a decrease in the levels of iNOS, IRF-7, Iba-1 and p65, as compared to LPS-treated mice. It is noteworthy that the same pattern of the CD36 mediated effects on TLR2-and TLR3 signalling were observed in human microglia, suggesting that lack of functional CD36 may lead to complete silencing of TLR2 and TLR3 downstream signalling and inflammation in immature mouse and human microglia. While CD36 acts as a co-receptor for TLR heterodimers and CD36/TLR2 signalling mediates injury in adult and neonatal stroke it also recognizes a plethora of ligands, such as low-density lipoprotein, long-chain fatty acids, thrombospondin-1, fibrillar β-amyloid (Aβ), and the cell membrane proteins of apoptotic cells^9,29–31^. CD36 is involved in angiogenesis, lipid metabolism, atherosclerosis, inflammation, phagocytosis, and clearing apoptotic cells^32,33^. In microglial cell cultures, CD36 is reported to act as an arbitrator of inflammatory mediators and ROS, thus suggesting its role in cytotoxicity^34–36^.

Interestingly, the effects were specific for TLR2 and TLR3 but not TLR4, as we observed that the TLR4-IRF3 pathway remained unaffected in mice as well in human microglial cell line, as depicted in form of a schematic in Fig. 6. IRF3 is an essential transcription factor involved in the non-MyD88, TRIF pathway following TLR4 activation^35^. Although TLRs activation is often viewed as pro-inflammatory, TLR4-IRF3 pathway is reported to have a dual role. Overexpression of IRF3 in primary human microglia is reported to alter the microglial inflammatory profile from the proinflammatory to the more anti-inflammatory by targeting the P13K/Akt pathway, thus further suggesting the existence of multiple molecular mechanisms that influence microglial immune profiles and control immune homeostasis^36^.

Taken together, our results showed that scavenger receptors like CD36 play an important role in the control of TLR2 and TLR3-mediated inflammation in neonatal brain. The TLRs-CD36 interactions control TLR2- mediated microglial activation and the consequent transcriptional regulation of cytokine and chemokine genes (Fig. 6a–c). Indeed, our data showed that disrupting CD36-TLR2 interaction has a significant impact on innate immune signalling in neonatal brain and ultimately on inflammation (Fig. 6b,c). Namely, we observed a general silencing of immune response together with downregulation of both pro and anti-inflammatory cytokines as well as different colony stimulating factors in the brains of P9 mice pre-treated with CD36 neutralizing in the context of LPS stimulation^37^. Of note, downregulation of pro-inflammatory cytokines, such as IL-1β, IL-6 and TNF-α, upon CD36 blockage, is also reported in PrP_106–126_ induced inflammation^38^.

Based on our results, we propose that following neonatal immune challenge (brain injury and/or infection), targeting scavenger receptors such as CD36 may serve as a promising molecular and/or therapeutic tool to curb TLR2 mediated inflammation and skew microglia immune signalling towards TLR4-IRF3 pathway, further suggesting its role in fine-tuning of neuroinflammation.

## Material and methods

### Mouse model

TLR2-luc-GFP transgenic mice were used to visualise the microglial activation/TLR2 induction and used as described before^4^. Transgenic animals were identified by polymerase chain reaction (PCR) detection of the luciferase transgene with the following primers: 5′-CAG-CAG-GAT-GCT-CTC-CAG-TTC-3′ and 5′-GGC-GCA-GTA-GGC-AAG-GTG-GT-3′. Genotyping was performed as previously described^4^. The pups were not weaned from their mother and were given ad libitum access to food and water and were housed with nesting material and shelters and kept in rooms with temperature control and light/dark cycles. All experimental procedures were approved by the Laval University animal care ethics committee (Protocol # 17-160-4) and are in accordance with The Guide to the Care and Use of Experimental Animals of the Canadian Council on Animal Care.

### LPS and anti-CD36 antibody injections

A schematic representation of the experimental protocol is illustrated in Fig. 1A. Unsexed TLR2-luc-GFP mice were intra-peritoneally (i.p.) injected anti-CD36 antibody (5 μg, mAb, Abcam, dissolved in 0.9% sterile saline) at P8 and LPS (1 mg/kg dissolved in 0.9% sterile saline) at P9. Control mice were injected with equal volume of sterile saline. Mice were then longitudinally imaged by in vivo bioluminescence for 3 days (n = 3)^16^.

### Tissue collection

The animals were anaesthetized by an i.p. injection of Ketamine/Xylazine (100/10 mg/kg) and transcardially perfused with 15 ml of phosphate buffered saline (PBS) 1×, followed by 4% paraformaldehyde (PFA) at pH 7.4 dissolved in PBS. Tissue samples were then post-fixed overnight in 4% PFA and equilibrated in PBS/30% sucrose for 48 h. Brains were embedded into Tissue-Tek (O.C.T. compound, Sakura, USA) and frozen at – 20 °C, cut into 15-µm thick coronal sections with Cryostat and stored at − 20 °C.

### Immunofluorescence

The brain sections were incubated overnight at room temperature using primary antibodies, as described in Table 1. After washes in PBS, the sections were incubated in corresponding fluorescent goat Alexa secondary antiserum (ThermoFisher, MA, USA). Fluorescent images of the cortical sections have been acquired using a Zeiss LSM 700 Confocal microscope with a 20× objective using a scan zoom between 1 and 2× and analysed with Zen software.

**Table 1:** List of antibodies for immunostaining.

Antibody	Dilution	Type	Company
Iba1	1:500	Rabbit polyclonal	Wako, Japan
MHC II	1:250	Rat monoclonal	AbD Serotec, UK
CD206	1:500	rat monoclonal	BioRad, USA
CD86	1:500	Rat monoclonal	BD Pharmingen, USA
GFAP	1:500	Rabbit polyclonal	Sigma Aldrich, USA

### Cytokine arrays

Protein expression analysis of inflammatory cytokines was performed using a mouse antibody array (RaybioMouse inflammation antibody array 1.1; #AAM-INF-1L; RayBiotech, Norcross, GA). Protein lysates were obtained by homogenization of saline perfused whole brains in 500 µl cell lysis buffer (included in the RayBiotech kit) with protease-inhibitor mixture (Complete Protease Inhibitor cocktail tablet, Roche, QC, Canada). Protein concentration was determined, and brain lysates were diluted to 300 μg in 1× blocking buffer. Samples from 3 to 5 mice/group were pooled and incubated with the array membrane overnight at 4 °C. After the washes, the membranes were incubated with the biotin-conjugated antibodies overnight at 4 °C. The membranes were then processed according to RayBiotech protocol. Membranes were exposed to X-ray film (Biomax light film; #1788207; Kodak) and analysed by ImageJ software.

### In vivo bioluminescence/biophotonic imaging

As previously described, the images were gathered using IVIS^®^ Spectrum Imaging System (PerkinElmer, Hopkinton MA, USA)^4,16^. Twenty-five minutes prior to the imaging session, the mice received i.p. injection of the luciferase substrate d-luciferine (150 mg/kg—of a solution of 20 mg/ml of d-luciferine dissolved in 0.9% saline was injected).

### Western blot analysis

Protein extracts were obtained from neonatal brains as described previously^16^. 500 mg of fresh brain tissue samples were transferred to 1 ml of cell lysis buffer (n = at least 3/group) (10 mM HEPES, 10 mM NaCl, 1 mM KH_2_PO_4_, 5 mM MgCl_2_) and homogenized by applying two strokes in a glass homogenizer. The suspension was incubated for 10 min on ice and then homogenized by applying six strokes in a motorized homogenizer at 250 rpm. The protein concentrations were determined by Bradford method. Protein samples were separated by electrophoresis on polyacrylamide gel and transferred on to a PVDF membrane. After blocking with 5% skimmed milk/5% BSA, the membranes were incubated overnight at 4 °C with primary antibodies as described in table below. After washes with PBS-Tween (0.1% Tween-20), the membranes were incubated with respective secondary antibodies conjugated with peroxidase (anti-goat/anti-mouse Jackson laboratory, USA). The membranes were washed in PBS-Tween and were processed for development using chemiluminescent reagent (Thermo pierce, USA) and exposed for different time periods on care stream Biomax Light or MR film (Kodak, NY, USA) or on BioRad ChemiDoc™ MP Imaging System. Densitometric analysis was analysed using Image J software. The membranes were stripped and re-probed with anti-GAPDH (Millipore, USA, 1:30,000) to determine loading of individual samples.

### Cell culture and western blot

HMC3 cells (ATTC, CRL-3304) were cultured in 10 ml DMEM with 10% FBS and antibiotic cocktail in 60 × 15 mm culture dishes (Sarstedt™). When the cells reached around 60–65% confluency, they were treated with either saline, or anti-CD36 blocking antibody (2 µg/ml), incubated for 18 h, followed by treatment with either LPS (1 µg/ml) or saline. The cells were further incubated for 24 h and later harvested as above for western blot. The cells were first washed with 5 ml DPBS, scraped and collected at 300 g, 10 min. The cell pellet was then washed with fresh 500 μL DPBS, centrifuged as above, and the pellet was collected for western blot (n = 3). The cells were incubated with NP40 lysis buffer on ice for 30 min, and centrifugation at 1200 rpm, 15 min at 4 °C. The supernatant was collected, and the protein quantification was done by Biorad DC kit. Western blot was done as described above. Blocking was done in 5% BSA, followed by treatment with respective primary antibodies (1:1000) and incubation at 4 °C overnight (Table 2). The membrane was washed with PBS-Tween (thrice, 10 min each) followed by addition of secondary antibodies (horse radish peroxidase conjugated-anti-mouse/anti-goat, Jackson Laboratory, 1:1000). The membranes were again washed in PBS-Tween and processed for development using chemiluminescent reagent (Thermo pierce, USA) and exposed for different time periods on care stream or on BioRad ChemiDoc™ MP Imaging System and results were analysed using Image j software. The membranes were stripped and re-probed with anti-GAPDH to determine equal loading of samples.

**Table 2:** List of antibodies for western blot.

Antibody	Dilution	Type	Company
Iba1	1: 1000	Rabbit polyclonal	Wako, Japan
TLR2	1: 1000	Rabbit polyclonal	Cell signaling, USA
TLR3	1: 1000	Rabbit polyclonal	ABCAM, UK
IRF7	1: 1000	Rabbit polyclonal	ABCAM, UK
p-P65	1: 1000	Rabbit monoclonal	Cell signaling, USA
TLR4	1: 1000	Rabbit polyclonal	Thermo Fischer Scientific, USA
IRF3	1: 1000	Rabbit polyclonal	EMD Millipore, USA
iNos	1: 1000	Rabbit polyclonal	Millipore, USA
GAPDH	1: 3000	Mouse monoclonal	Santa Cruz Biotechnology, USA

### Statistical analysis

All data are presented as mean ± SEM. Unpaired t test with Welch's correction was performed for quantifying immunostaining data. Unpaired t-test was performed for the cytokine array. Statistical analysis for the western blots was performed for the four data sets by two-way ANOVA followed by Tukey’s multiple comparisons test between the groups control vs. LPS, control vs. anti-CD36, and LPS vs. anti-CD36 + LPS. ****p < 0.0001; ***p ≤ 0.001; **p ≤ 0.01 and *p ≤ 0.05.

We confirm that the study is reported in accordance with ARRIVE guidelines.

### Ethical approval

All the experimental procedures were approved by the Laval University Animal Care Ethics Committee under the approved protocol # 17-160-4 and are in accordance with the *Guide to the Care and Use of Experimental Animals* of the Canadian Council on Animal Care.

## Supplementary Information

Supplementary Information.

## Data Availability

All data generated during this study are included in this article or are available on reasonable request from the corresponding authors.
