# Supplementary material for: CD36 neutralisation blunts TLR2-IRF7 but not IRF3 pathway in neonatal mouse brain and immature human microglia following innate immune challenge
Source: Sci Rep. 2023 Feb 9;13:2304. doi: 10.1038/s41598-023-29423-0 (PMC9911392; doi:10.1038/s41598-023-29423-0)
Supplement: Supplementary file 1 — Supplementary Information. [file 41598_2023_29423_MOESM1_ESM.pdf]

Supplementary material

Fig. 1: Cytokine array membranes

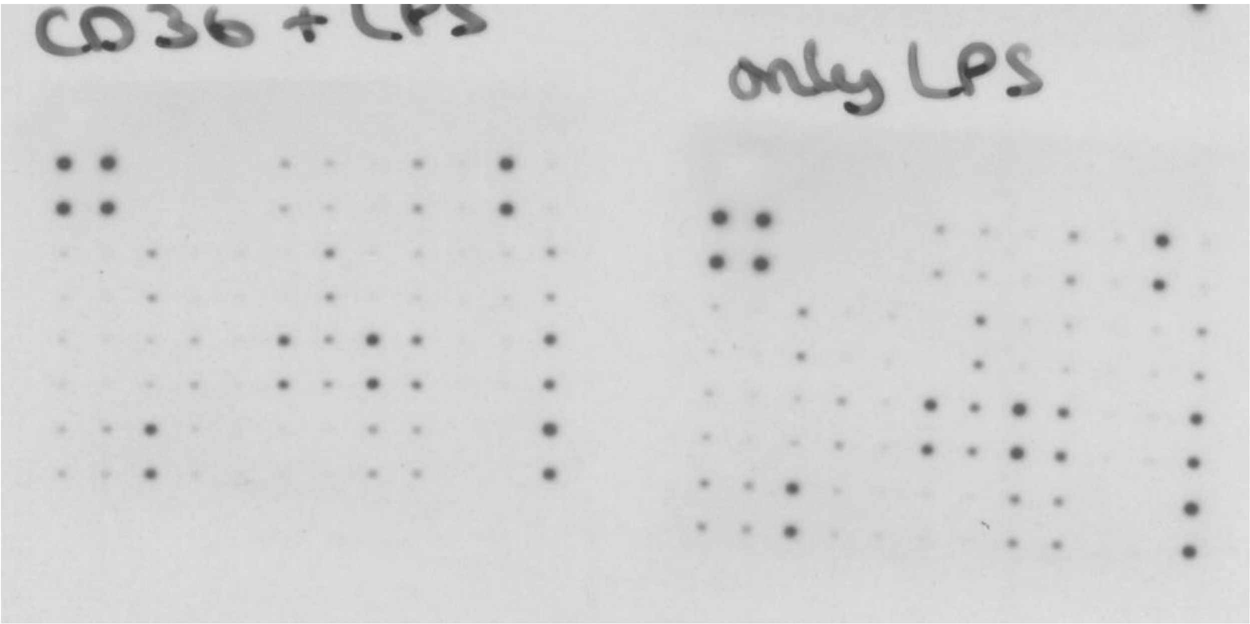

Each antibody is spotted in duplicate vertically

	A	B	C	D	E	F	G	H	I	J	K	L
1 2	POS	POS	NEG	NEG	BLANK	BLC (CXCL13)	CD30 Ligand (TNFSF8)	Eotaxin-1 (CCL11)	Eotaxin-2 (CCL24)	Fas Ligand (TNFSF6)	Fractalkine (CX3CL1)	GCSF
3 4	GM-CSF	IFN-gamma	IL-1 alpha (IL-1 F1)	IL-1 beta (IL-1 F2)	IL-2	IL-3	IL-4	IL-6	IL-9	IL-10	IL-12 p40/p70	IL-12 p70
5 6	IL-13	IL-17A	I-TAC (CXCL1)	KC (CXCL1)	Leptin	LIX	XCL1	MCP-1 (CCL2)	M-CSF	MIG (CXCL9)	MIP-1 alpha (CCL3)	MIP-1 gamma
7 8	RANTES (CCL5)	SDF-1 alpha (CXCL12 a)	I-309 (TCA-3/CCL1)	TECK (CCL25)	TIMP-1	TIMP-2	TNFalpha	TNF RI (TNFRSF1A)	TNF RII (TNFRSF1B)	BLANK	BLANK	POS

Fig. 2: Blots for western blot fig 4

Blots for Iba-1 and TLR-2 were cut pre-hybridisation.

Similarly blots for TLR3 and IRF7 were cut and TLR-4 and p-p65 were cut.

Iba-1

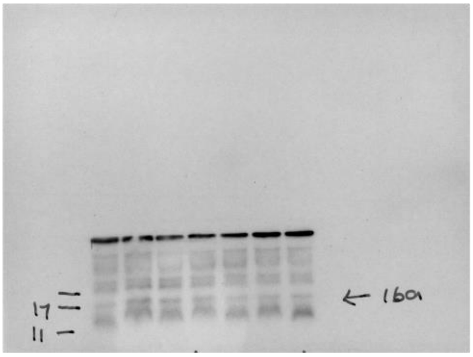

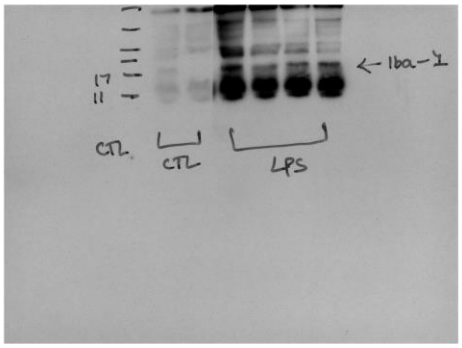

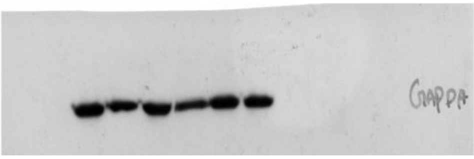

TLR2

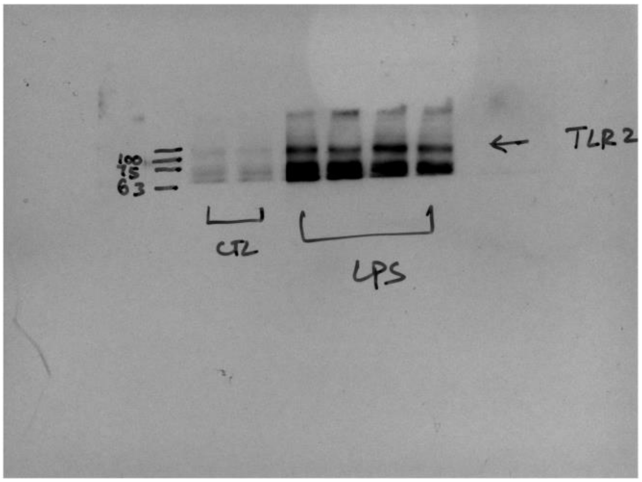

TLR3

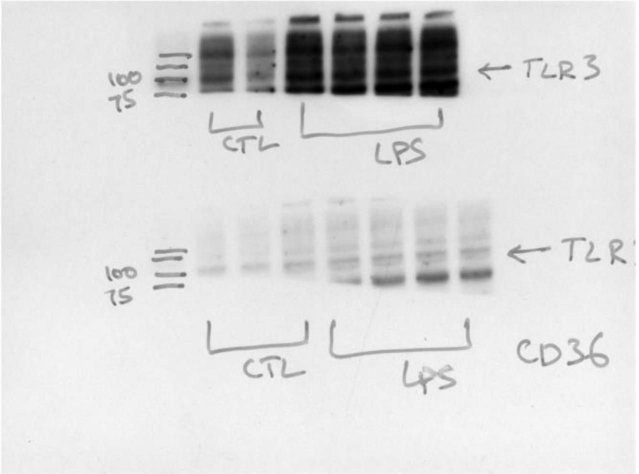

GAPDH

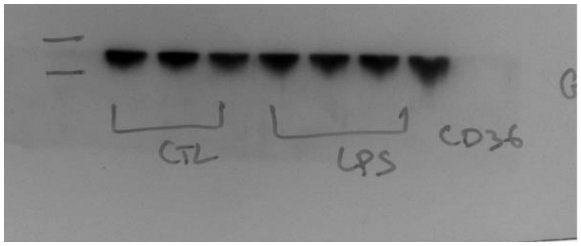

IRF7

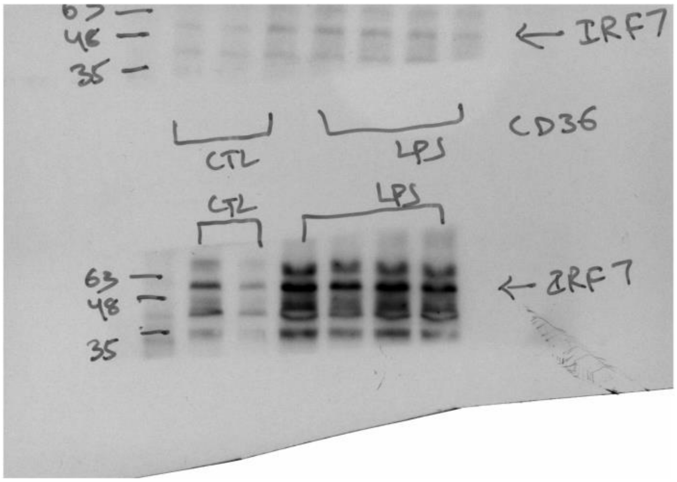

IRF3 (rev)

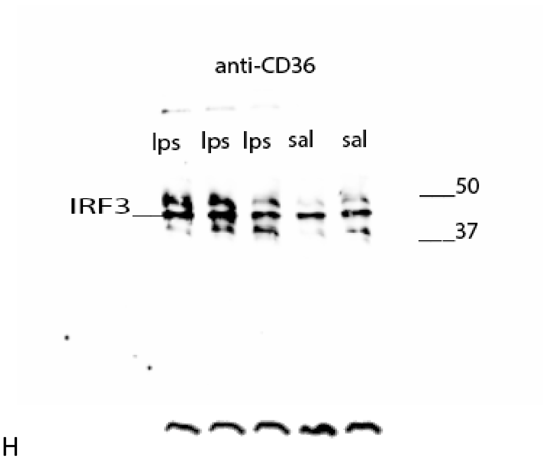

GAPDH

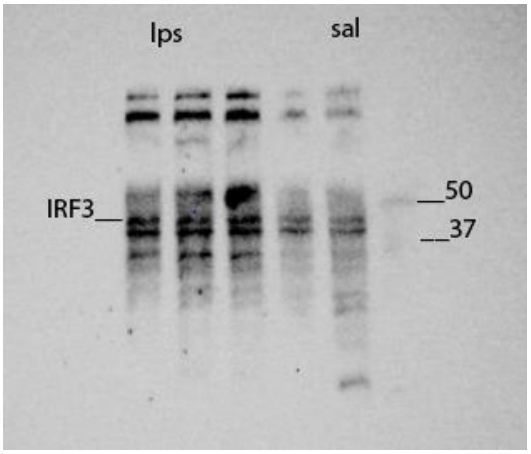

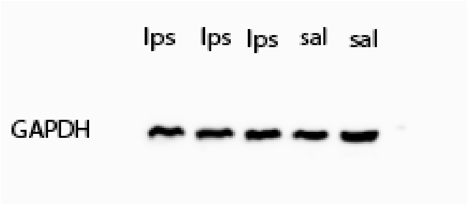

IRF3

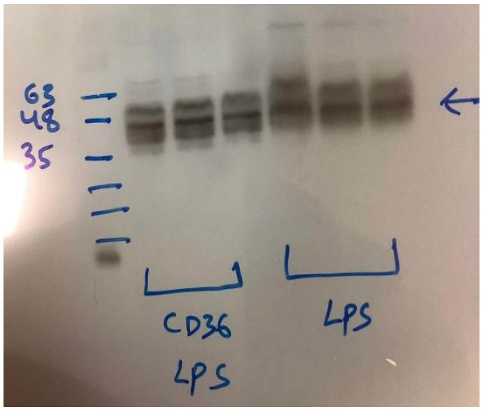

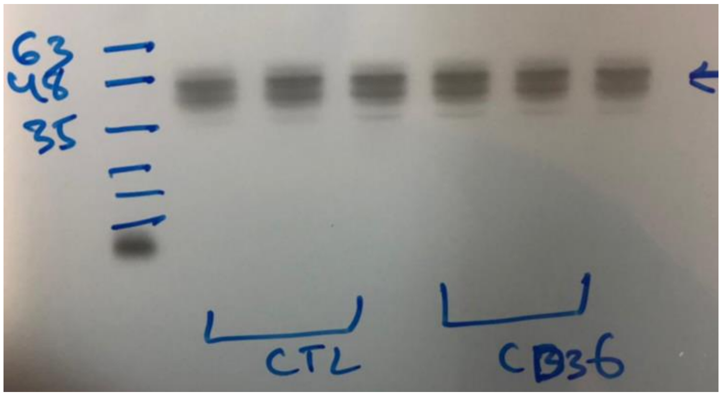

GAPDH for IRF3

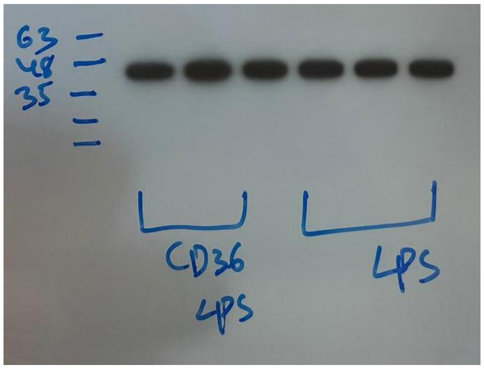

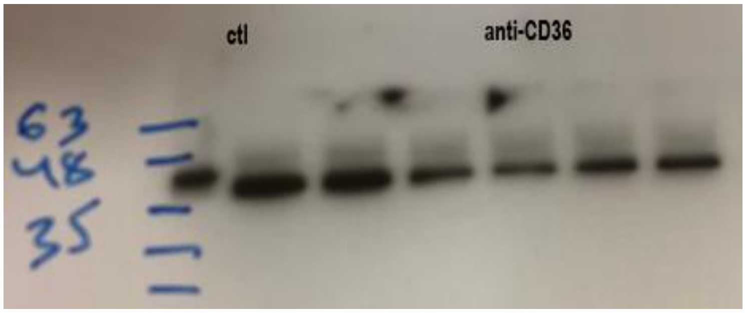

Inos

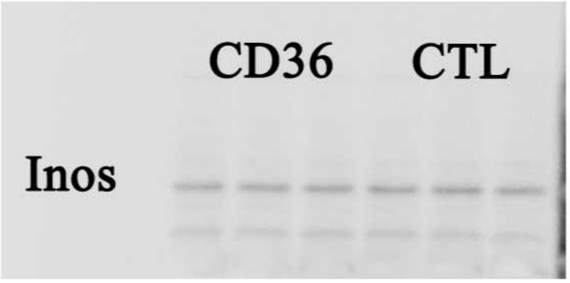

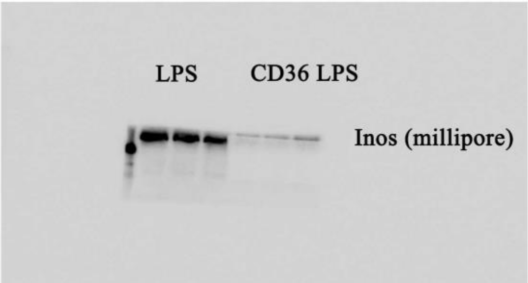

P-p65

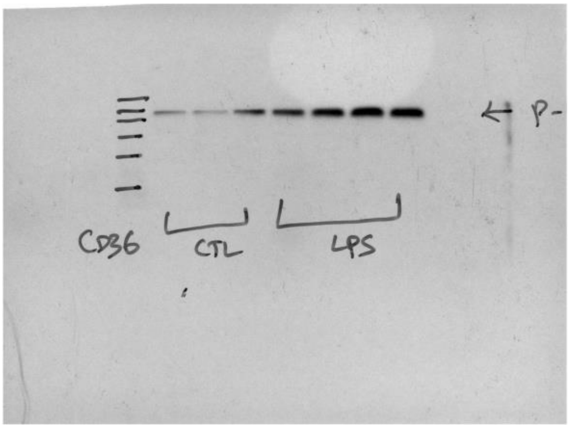

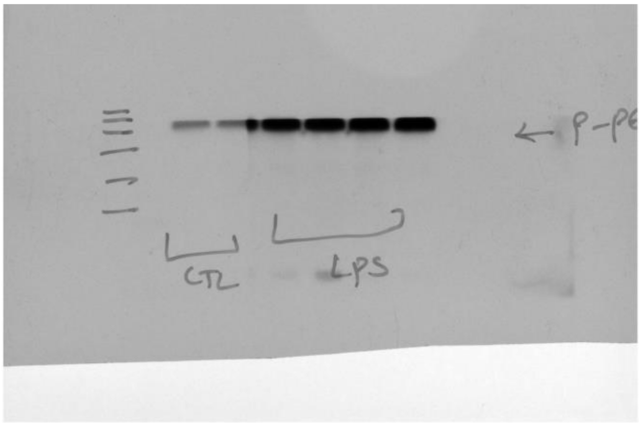

GAPDH for P-p65

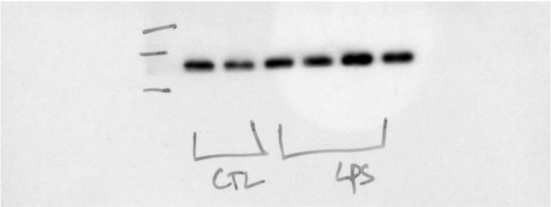

TLR4

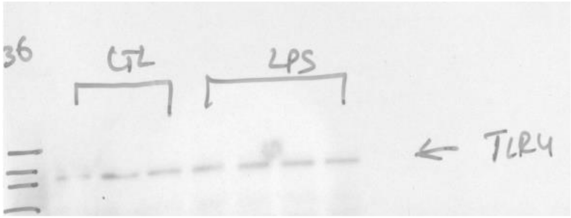

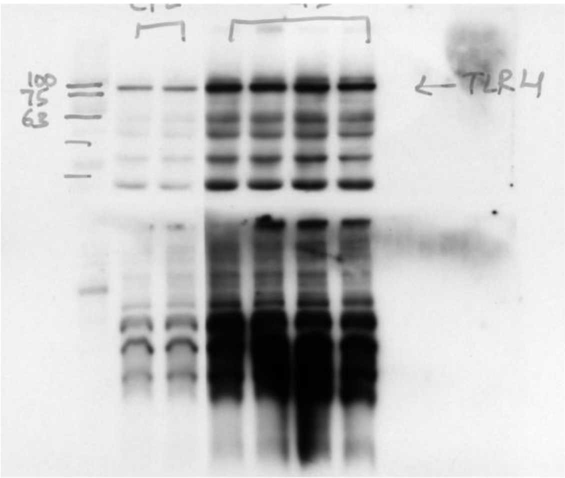

GAPDH for TLR4 and p-p65

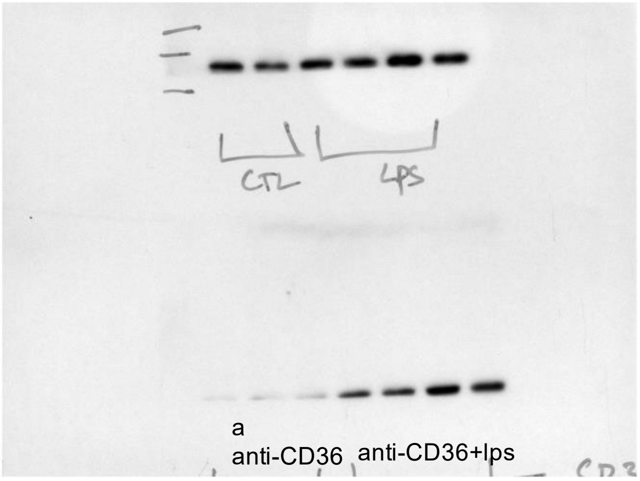

Fig. 3: Blots for figure 5 HMC lysates

Membranes for Iba-1 and TLR3 (anti-CD36, anti-CD36+LPS), were cut post-transfer.

Similarly, membranes for TLR2, p65 (ctl, lps), TLR 4 and IRF7 (anti-cd36, anti-cd36+lps)

IRF7 and TLR3 (ctl, lps) were prepared.

The blots were analysed on BioRad ChemiDoc at various exposure times.

Iba-1

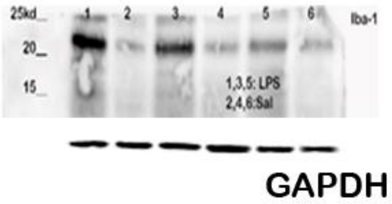

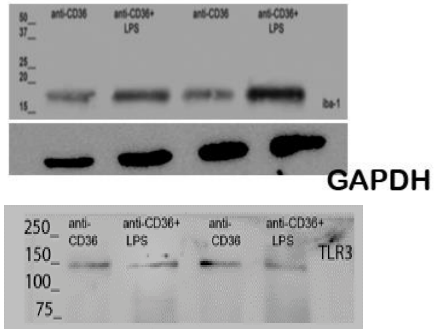

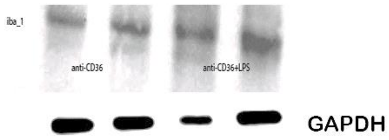

TLR 3

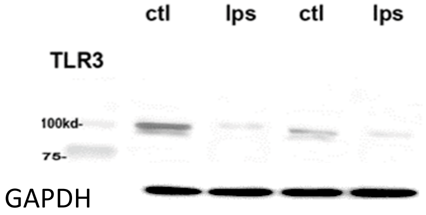

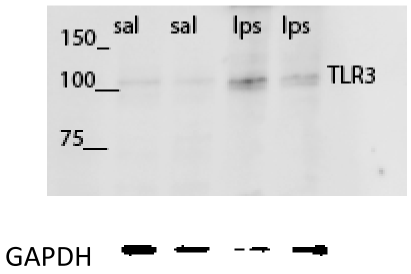

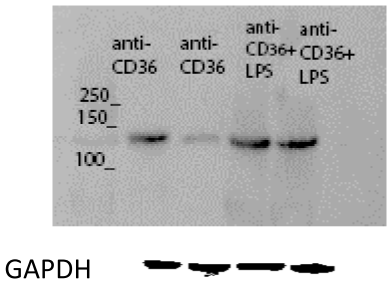

TLR-2

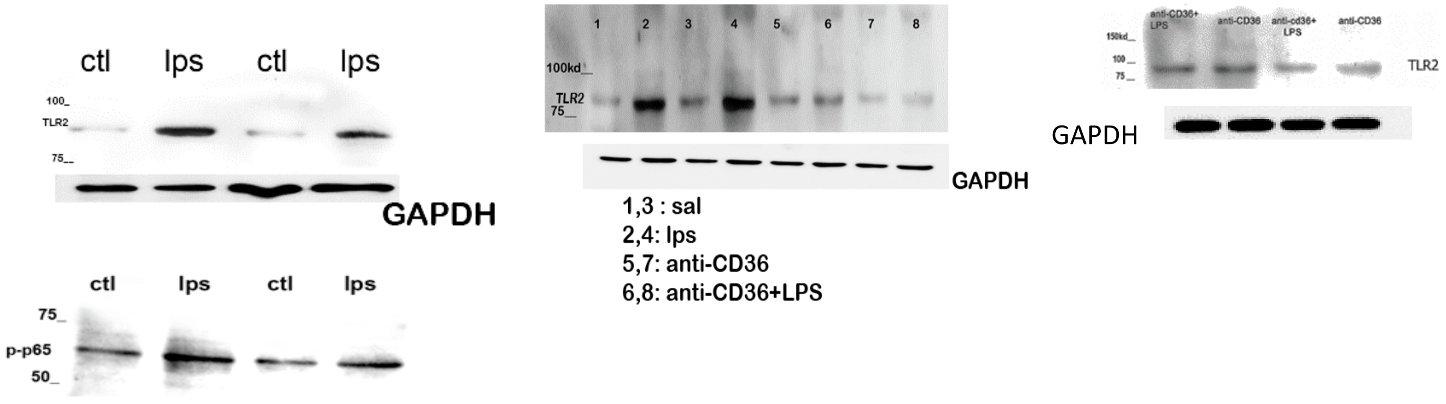
ctl lps ctl lps
100_
TLR2
75_
GAPDH
ctl lps ctl lps
75_
p-p65
50_
1 2 3 4 5 6 7 8
100kd
TLR2
75
GAPDH
1,3 : sal
2,4: lps
5,7: anti-CD36
6,8: anti-CD36+LPS
anti-CD36+ LPS
anti-CD36
anti-cd36+ LPS
anti-CD36
150kd
100
75
TLR2
GAPDH

IRF-7

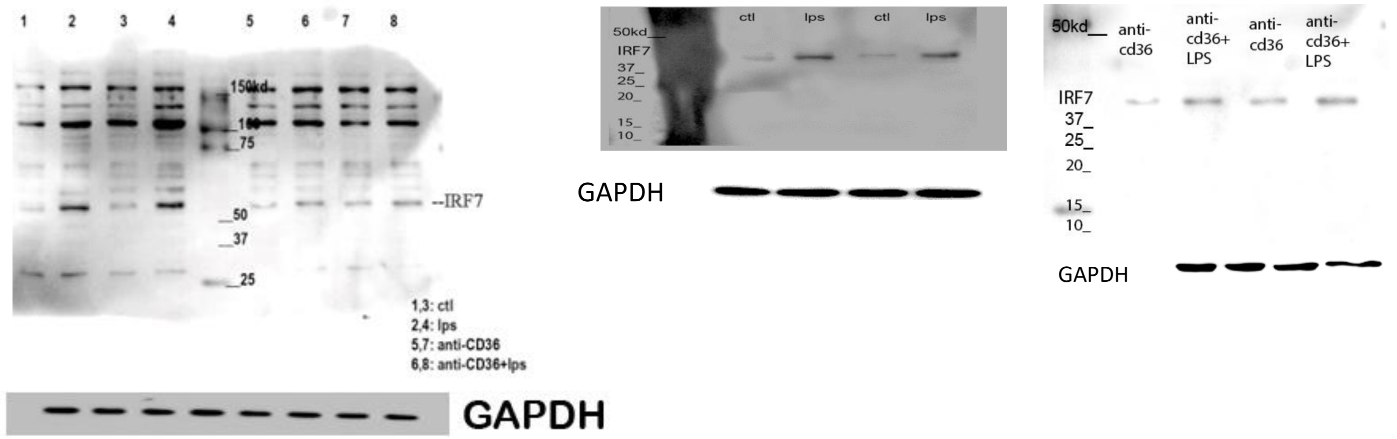
1 2 3 4 5 6 7 8
150kd
100
75
50
37
25
IRF7
1,3: ctl
2,4: lps
5,7: anti-CD36
6,8: anti-CD36+lps
GAPDH
ctl lps ctl lps
50kd
IRF7
37
25
20
15
10
GAPDH
50kd
anti-cd36
anti-cd36+ LPS
anti-cd36
anti-cd36+ LPS
IRF7
37
25
20
15
10
GAPDH

IRF3

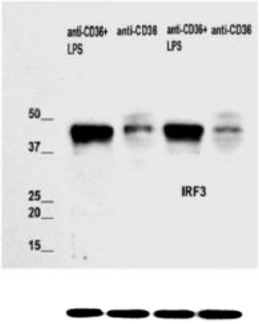

GAPDH

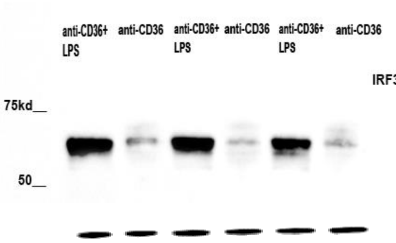

GAPDH

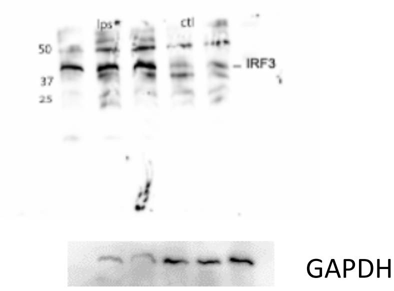

GAPDH

TLR4

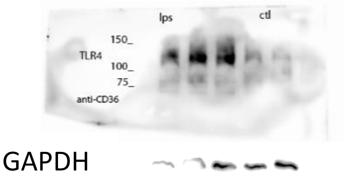

GAPDH

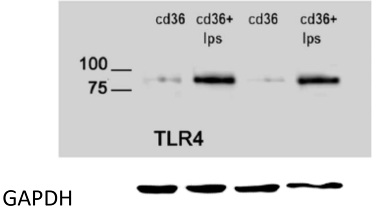

GAPDH

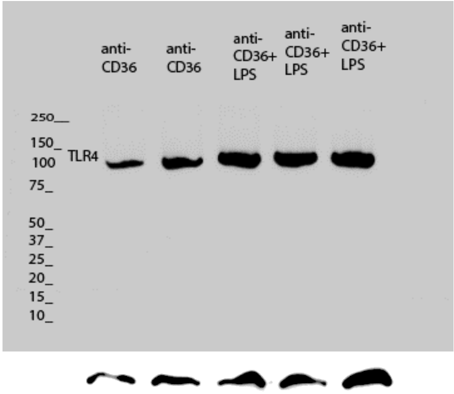

GAPDH

Inos

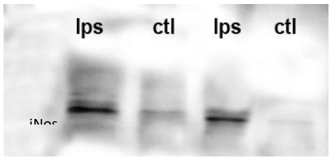

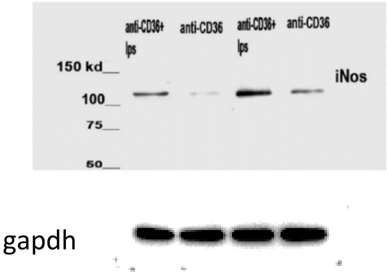

gapdh
